# Health-Related Quality of Life Among Members Using an On Demand Behavioral Health Platform: Pilot Observational Study

**DOI:** 10.2196/35352

**Published:** 2022-07-08

**Authors:** Emily Shih, Brandon S Aylward, Sarah Kunkle, Grant Graziani

**Affiliations:** 1 Ginger San Francisco, CA United States

**Keywords:** behavioral coaching, mental health, telehealth, Healthy Days, clinical care, behavior, coach, quality of life, platform, tool, pilot study, observational, health-related quality of life, virtual care, association, text-based, outcome, evaluation

## Abstract

**Background:**

Despite the well-known adverse health conditions and negative economic outcomes associated with mental health problems, accessing treatment is difficult due to reasons such as availability and cost. As a solution, digital mental health services have flooded the industry, and new studies are quickly emerging that support their potential as an accessible and cost-effective way to improve mental health outcomes. However, many mental health platforms typically use clinical tools such as the Patient Health Questionnaire-9 (PHQ-9) or General Anxiety Disorder-7 (GAD-7). Yet, many individuals that seek out care do not have clinical symptomatology and thus, traditional clinical measures may not adequately capture symptom improvement in general well-being. As an alternative, this study used the health-related quality of life (HRQoL) tool from the Centers for Disease Control and Prevention “Healthy Days” measure. This subjective measure of well-being is an effective way to capture HRQoL and might be better suited as an outcome measure for treatments that include both clinical and subclinical individuals.

**Objective:**

The purpose of this study was to describe changes in HRQoL in clinical and subclinical members assessing virtual care and to examine the association between text-based behavioral coaching and virtual clinical sessions with changes in HRQoL.

**Methods:**

A total of 288 members completed the 4-item HRQoL measure at baseline and at 1 month following use of the Ginger on demand behavioral health platform. Baseline anxiety and depression levels were collected using the GAD-7 and PHQ-9, respectively.

**Results:**

Members completed on average 1.92 (SD 2.16) coaching sessions and 0.91 (SD 1.37) clinical sessions during the assessment month. Paired samples *t* tests revealed significant reductions in the average number of unhealthy mental health days between baseline (mean 16, SD 8.77 days) and follow-up (mean 13.2, SD 9.02 days; *t*_287_=5.73; *P*<.001), and in the average number of days adversely impacted (mean_baseline_ 10.9, mean*_follow-up_* 8.19; *t*_287_=6.26; *P*<.001). Both subclinical members (*t*_103_=3.04; *P*=.003) and clinical members (*t*_183_=5.5; *P*<.001) demonstrated significant improvements through reductions in adversely impacted days over a month. Clinical members also demonstrated significant improvements through reductions in unhealthy mental health days (*t*_183_=5.82; *P*<.001). Finally, member engagement with virtual clinical sessions significantly predicted changes in unhealthy mental health days (*B*=–0.96; *P*=.04).

**Conclusions:**

To our knowledge, this study is one of the first to use the HRQoL measure as an outcome in an evaluation of a digital behavioral health platform. Using real-world longitudinal data, our preliminary yet promising results show that short-term engagement with virtual care can be an effective means to improve HRQoL for members with subclinical and clinical symptoms. Further follow-up of reported HRQoL over several months is needed.

## Introduction

Nearly 1 in 5 adults in the United States (51.5 million people) experience mental health issues [1]. The World Health Organization estimates that anxiety and depression alone cost the global economy US $1 trillion dollars each year in lost productivity, absenteeism, and medical costs [2]. Mental health issues have been exacerbated with the recent COVID-19 pandemic and underscore a critical moment of global need [3,4]. A recent meta-analysis found the global prevalence of diagnosable anxiety and depression during the pandemic was 27% and 28%, respectively [5]. Even among those with subclinical symptoms, nearly half of adults in the United States have reported symptoms of anxiety or depression during this time [6]. Timely intervention for those with subclinical symptoms is just as important to prevent development of more serious symptoms requiring more costly treatment.

Despite the well-known adverse health conditions and negative economic outcomes, accessing treatment for common mental health problems is difficult [7]. The demand for mental health services has outpaced the availability of qualified mental health professionals. A recent survey found that 1 in 4 individuals with depression or anxiety lack access to care or have unmet mental health needs [8]. In addition, long wait lists, high out-of-pocket expenses, and transportation burdens all continue to serve as barriers to receipt of effective services [9,10]. There is a growing need for scalable mental health solutions that increase both the availability of professionals and access to care for common mental health conditions. This is particularly important with the recent increase of mental health issues during the pandemic. Digital mental health services have flooded the industry, and new studies are emerging that support their potential to serve as cost-effective ways to manage anxiety and depression [11,12]. This type of support can even be beneficial for individuals who may be at risk for but do not yet experience clinically significant symptoms [13].

Many mental health platforms typically use clinical tools such as the Patient Health Questionnaire-9 (PHQ-9) or General Anxiety Disorder-7 (GAD-7) for assessing initial and treatment outcomes of depressive and anxiety symptoms, respectively. As behavioral coaching focuses on goal-oriented behavior and typically targets those with subclinical symptomatology, traditional clinical measures may not adequately capture symptom improvement in general mental health and well-being. State and federal health agencies have supported the population surveillance of health-related quality of life (HRQoL), which is a multidimensional concept that examines overall health related to perceived physical and mental health as well as daily functioning [14,15]. One common HRQoL tool is the Centers for Disease Control and Prevention (CDC) “Healthy Days” measure that asks about self-rated general health, physical health, mental health, and activity limitations over the past 30 days. This subjective measure of well-being is an effective way to capture HRQoL and might be better suited as an outcome measure for treatments that include both clinical individuals and individuals with symptoms not meeting clinical thresholds [16]. Yet, few studies have used this measure when evaluating digital behavioral health platforms. Financially, Humana found that the cost of each reported unhealthy day is equivalent to 10 hospital admissions per thousand patients, with a potential increase of US $15.64 per member per month in medical costs for each unhealthy day [17]. This highlights the potential long-term savings that could result from interventions targeting individual HRQoL. A previous health coaching study has already demonstrated significant reductions in reported unhealthy days among participants [18].

The purpose of this study was to examine self-reported HRQoL among members using an on demand digital health platform and the association of short-term text-based behavioral health coaching and virtual clinical sessions with healthy days over time. To that end, the study will describe baseline characteristics of members in terms of reported unhealthy days and changes over 1 month, describe changes in unhealthy days as a function of baseline anxiety and depressive symptoms, and examine the association between member engagement and changes in unhealthy days.

## Methods

### Participants

Participants were members who had access to the Ginger on demand behavioral health platform as part of their employer or health plan benefits. Internal clinical protocols include exclusionary criteria where self-directed telehealth is likely not appropriate and where more specialized and urgent psychiatric services are required (eg, active suicide ideation or active high-risk self-harm behavior; see Kunkle et al [19] for exhaustive list). This study included Ginger members 18 years or older who completed the baseline Healthy Days measure between November 2020 to November 2021 and who first accessed care within 1 month of completing their Healthy Days baseline.

### Procedures

The Ginger platform provides members with access to virtual behavioral health coaching, teletherapy, telepsychiatry, and self-guided content and assessments, primarily via a mobile app platform. After downloading the mobile app, members can start texting with a behavioral health coach within minutes of requesting to connect. Ginger coaches are full-time employees who have an advanced degree in a field related to mental health or have accredited coach certification. While many members are solely engaged with text-based coaching services, some will request or require escalation to clinical services (teletherapy or telepsychiatry) depending on preference or clinical severity. When members are escalated to therapy or psychiatry, they may continue working with a coach provided they also seek additional specialized care concurrently. Additional detail regarding Ginger can be found in prior publications [19,20].

The Healthy Days measure was administered to members 4 times across the span of 4 months (once per month). Data were collected externally using the Survey Monkey platform. Only responses from survey items pertaining to the number of unhealthy mental health days and impacted days were of focus for this study. The PHQ-9 and GAD-7 were typically completed at intake within 1 month of the Healthy Days baseline assessment.

### Measures

The CDC Healthy Days measure contains four items: (1) “Would you say that in general your health is excellent, very good, good, fair, or poor?” (2) “Now thinking about your physical health, which includes physical illness and injury, for how many days during the past 30 days was your physical health not good?” (3) “Now thinking about your mental health, which includes stress, depression, and problems with emotions, for how many days during the past 30 days was your mental health not good?” and (4) “During the past 30 days, for about how many days did poor physical or mental health keep you from doing your usual activities, such as self-care, work, or recreation?” (referred to here as impacted days). For this study a change variable was calculated by subtracting reported unhealthy scores from time 1 from scores from time 2, where positive values indicate an increase in unhealthy days, whereas negative values indicate a reduction in unhealthy days.

The PHQ-9 is a 9-item self-report questionnaire that assesses the frequency and severity of depression symptomatology within the previous 2 weeks. Each of the 9 items is based on the *Diagnostic and Statistical Manual of Mental Disorders* (Fourth Edition; *DSM-IV*) criteria for major depressive disorder and are scored on a 0 (not at all) to 3 (nearly every day) scale. Items include “Little interest or pleasure in doing things” and “Feeling down, depressed, or hopeless.” Total scores can range from 0 to 27 with higher scores indicating more depressive symptoms. A score of 10 was used as the clinical threshold [21].

The GAD-7 is a valid brief self-report tool to assess the frequency and severity of anxious thoughts and behaviors over the past 2 weeks. Each of the 7 items are based on the *DSM-IV* diagnostic criteria for generalized anxiety disorder and are scored on a 0 (not at all) to 3 (nearly every day) scale, with total scores ranging from 0 to 21. Items include “Feeling nervous, anxious, or on edge” and “Not being able to stop or control worrying.” Consistent with existing literature, a score of 10 was used as the clinical threshold [22].

Member engagement with Ginger services was quantified as the number of coaching and clinical sessions. Coaching sessions were operationalized as the number of unique days where both members and coaches each sent at least 5 text messages. Ginger coaching is an on demand text-based service, and the operationalization of a “text-based coach session” has not been predetermined in the literature. As such, our threshold was decided based upon internal work that highlighted approximately 5 texts each way as the number of text messages needed to capture a productive conversation between members and their coaches. Clinical sessions were operationalized as the number of completed video sessions with a clinician.

### Statistical Analysis

Analyses were conducted using RStudio (version 1.4.1717; RStudio, PBC). Data were first screened for outliers and normality. Descriptive statistics were used to describe baseline member characteristics. For changes in reported unhealthy days, paired sample *t* tests were used. Next, members were divided into groups as a function of clinical thresholds using the PHQ-9 and GAD-7 scores at intake (ie, clinical vs subclinical). Additional paired sample *t* tests were performed to evaluate member differences in responses between time 1 and time 2 for clinical and subclinical groups separately. A Benjamini-Hochberg correction was used to adjust for multiple comparisons [23]. Finally, scatterplots suggested a linear trend between member engagement and changes in unhealthy days. As such, multiple linear regressions were performed to examine the association of member engagement (ie, coaching and clinical sessions) with changes in the number of unhealthy days. Baseline Healthy Days scores and the number of prior engagement levels were entered as covariates. All continuous variables were standardized for interpretability.

### Ethics Approval

This is a secondary analysis of pre-existing deidentified data. The authors do not have access to participant identifying information and do not intend to recontact participants. Ginger’s research protocols and supporting policies have been reviewed and approved by Advarra’s institutional review board (Pro00046797) in accordance with the US Department of Health and Human Services regulations at 45 CFR 46.

## Results

### Descriptive Statistics

A total of 1496 members completed the Healthy Days measure at time 1 (intake), 351 (23.5%) members at time 2 (~30 days following intake; mean 31.9, SD 1.48 days), 114 members at time 3 (~60 days following intake), and 37 members at time 4 (~90 days following intake). The current analyses examined only members who had completed surveys at both time 1 (intake) and at time 2 (N=288). Data were missing at random for all primary outcome variables (*t*>–0.70 and *t*<1.54; *P*>.12). Potential reasons for earlier drop-offs that should be taken into consideration when interpreting our results include members having achieved their coaching goals, members no longer interested in care, and members engaged at a monthly cadence and returned after the study evaluation month was finished. Demographic information about members was provided by employers but contained missing data. Of members in the analytical sample, 82 (28.5%) members were between the ages of 18-34 years, 96 (33.3%) members were 35 years of age or older, and 110 (38.2%) members did not have age reported. Regarding gender identity, 125 (43.4%) members identified as female, 33 (11.5%) as male, 14 (4.9%) as other, and 116 (40.3%) did not have gender reported.

**Figure 1:**
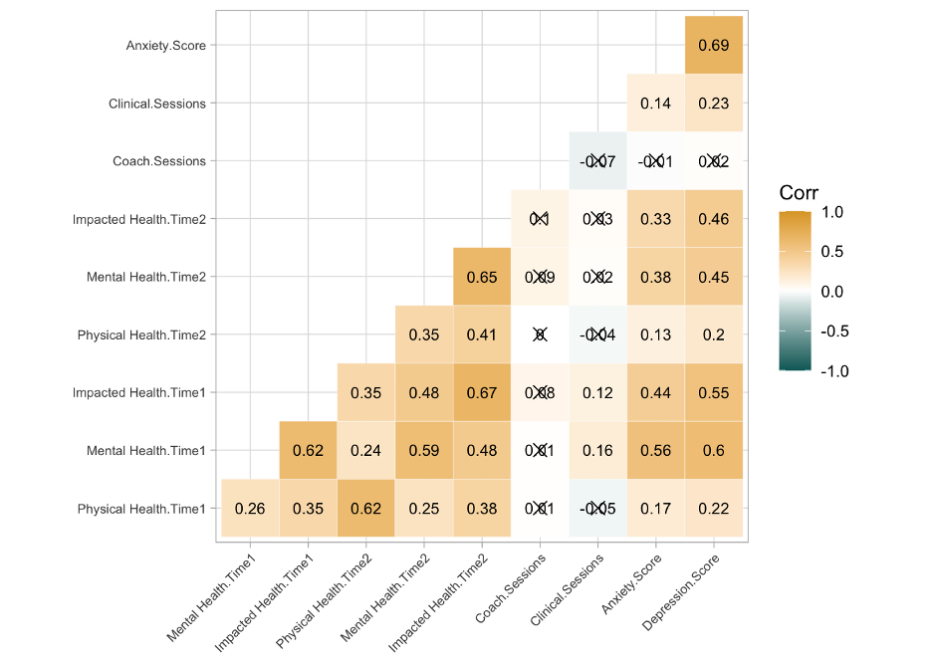
Correlations among primary variables. Note: Insignificant correlations where *P*>.05 are marked. Corr: correlation.

Descriptive statistics for the primary variables are presented in Table 1. Members, on average, completed 1.92 (SD 2.16, range 0-12) coaching sessions and 0.91 (SD 1.37, range 0-5) video sessions with a clinician within a single month. A total of 179 (62.2%) members engaged exclusively with text-based coaching (no clinical sessions). Subclinical depression and anxiety levels were reported in 104 (36.1%) members, whereas 184 (63.9%) members reported clinical levels of depression or anxiety. Of members in the analytical sample, 71% (n=205) at time 1 and 77% (n=223) of members at time 2 reported feeling “good” or better in response to the question “Would you say that in general your health is excellent, very good, good, fair, or poor?” (includes members who reported feeling “very good” and “excellent”). Bivariate correlations among the primary variables are presented in Figure 1. Of note, the number of unhealthy mental health days was positively correlated with the number of impacted health days at each respective time point (*r*=0.62 at time 1, *r*=0.65 at time 2; *P*<.001).

**Table 1:** Descriptive statistics among primary variables.

	Values, mean (SD)	Min	Max
Physical health (time 1)	5.1 (7.8)	0	30
Mental health (time 1)	16.0 (8.8)	0	30
Impacted health (time 1)	10.9 (9.6)	0	30
Physical health (time 2)	5.6 (8.3)	0	30
Mental health (time 2)	13.2 (9.0)	0	30
Impacted health (time 2)	8.2 (8.5)	0	30
Coaching sessions	1.9 (2.2)	0	12
Clinical sessions	0.9 (1.4)	0	5
Depression score (PHQ-9^a^)	11.3 (6.1)	1	27
Anxiety Score (GAD-7^b^)	9.8 (5.7)	0	21

^a^PHQ-9: Patient Health Questionnaire-9.

^b^GAD-7: General Anxiety Disorder-7.

### Pre-Post Changes in Reported Unhealthy Days

**Figure 2:**
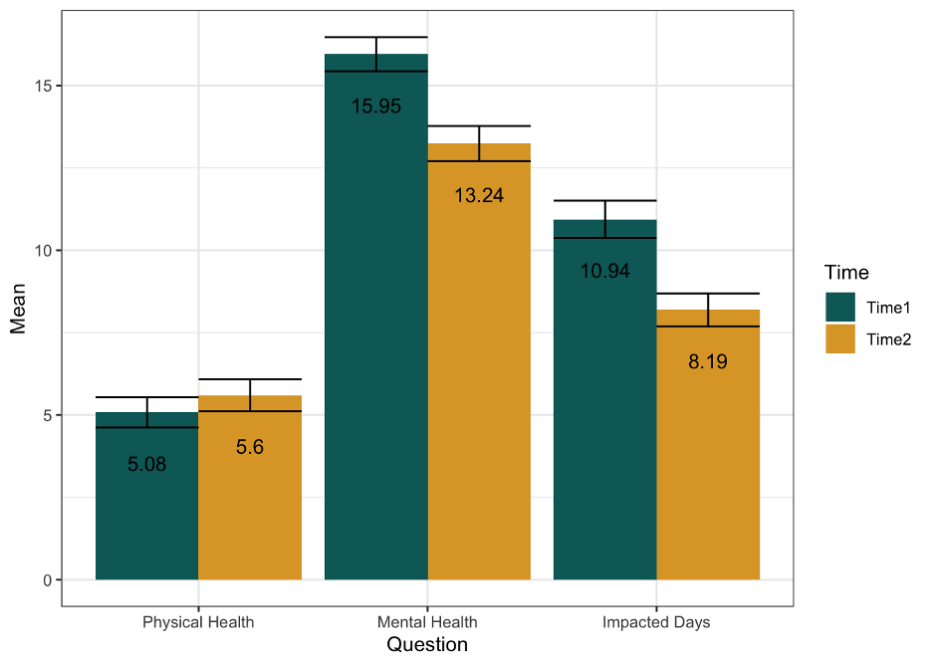
Display of means across the items from the Healthy Days measure at time 1 and time 2 (N=288).

Members reported on average nearly 3 fewer unhealthy mental health days (mean –2.71, SD 8.03) between baseline and 1 month later. Of the analytical sample, 61% (n=175) of members reported an improvement in unhealthy mental health days, whereas 39% (n=113) reported no improvement or an increase in unhealthy mental health days. Paired sample *t* tests were performed to evaluate differences in member Healthy Days responses at time 1 compared to time 2 (Figure 2) across all continuous items. Results showed no significant improvements in unhealthy physical health days between time 1 (mean 5.08, SD 7.78 days) and time 2 (mean 5.60, SD 8.25 days; *t*_287_=–1.25; *P*=.21). However, results showed significant improvements in unhealthy mental health days between time 1 (mean 16, SD 8.77 days) and time 2 (mean 13.2, SD 9.02 days; *t*_287_=5.73; *P*<.001), as well as significant improvements in adversely impacted days between time 1 (mean 10.9, SD 9.60 days) and time 2 (mean 8.19, SD 8.51 days; *t*_287_=6.26; *P*<.001). Given Ginger is a mental health platform and significant changes were only observed for unhealthy mental health days and adversely impacted days, these two outcomes were explored in subsequent analyses.

### Comparison of Change in Healthy Days Between Clinical and Subclinical Members

Subclinical members showed trending reductions in reported unhealthy mental health days between time 1 (mean 9.92, SD 6.78 days) and time 2 (mean 8.44, SD 7.83 days; *t*_103_=1.87; *P*=.06, adjusted *P*=.06). Clinical members also showed reductions in reported unhealthy mental health days between time 1 (mean 19.4, SD 7.91 days) and time 2 (mean 16.0, SD 8.52 days; *t*_183_=5.82; *P*<.001; adjusted *P*<.001).

Similarly, subclinical members showed significant reductions in reported impacted days at time 1 (mean 5.15, SD 6.64 days) compared to time 2 (mean 3.47, SD 5.3 days; *t*_103_=3.04; *P*=.003, adjusted *P*=.003). Clinical members also showed significant reductions in reported impacted days at time 1 (mean 14.2, SD 9.48 days) compared to time 2 (mean 10.9, SD 8.83 days; *t*_183_=5.50; *P*<.001, adjusted *P*=.001).

### Member Engagement on Changes in Reported Unhealthy Mental Health Days

The linear regression model predicting changes in reported unhealthy mental health days was significant (*F*_5,282_=14.6; *P*<.001) and accounted for 21% of the variance. No significant main effects of coaching sessions (*B*=0.61; *P*=.19) were observed. However, there was a significant main effect of clinical sessions (*B*=–0.96; *P*=.04), where more clinical sessions was associated with a decrease in unhealthy mental health days. The model predicting changes in adversely impacted days was also significant (*F*_5,282_=22.2; *P*<.001) and accounted for 28% of the variance. No significant main effects of coaching sessions (*B*=0.43; *P*=.30) or clinical sessions (*B*=–0.40; *P*=.33) were observed. Coefficients for both models are presented in Table 2.

**Table 2:** Summary of regression coefficients (N=288).

			Beta (SE)		*P* value
**Model 1: Changes in the number of unhealthy mental health days**					
	(Intercept)	–2.71 (0.43)		<.001	
	Unhealthy mental health days (baseline)	–3.32 (0.43)		<.001	
	Prior coaching sessions	0.39 (0.47)		.42	
	Prior clinical sessions	0.59 (0.47)		.21	
	Clinical sessions	–0.96 (0.47)		.04	
	Coaching sessions	0.61 (0.47)		.19	
**Model 2: Changes in the number of adversely impacted days**					
	(Intercept)	–2.75 (0.38)		<.001	
	Unhealthy impacted days (baseline)	–3.87 (0.38)		<.001	
	Prior coaching sessions	–0.30 (0.42)		.48	
	Prior clinical sessions	0.10 (0.41)		.80	
	Clinical sessions	–0.40 (0.41)		.33	
	Coaching sessions	0.43 (0.41)		.30	

## Discussion

### Principal Findings

This study evaluated the real-world association between digital care utilization in members with both subclinical and clinical symptoms of anxiety or depression. HRQoL at baseline suggested that members were, on average, demonstrating “frequent distress” and reporting more *unhealthy* mental health days than *healthy* mental health days (mean 16, SD 8.77 days; 53% of the month). The CDC defines having ≥14 unhealthy mental health days as “frequent distress [24].” Of note, our results also observed a relatively high number of unhealthy mental health days (mean 9.92, SD 6.78 days; 33% of the month) for subclinical members at baseline, highlighting the need for care for those that might not traditionally be recommended for clinical services (eg, individuals who might not have exceeded clinical thresholds using traditional PHQ-9 and GAD-7 assessment surveys). Bivariate correlations revealed a positive association between unhealthy mental health days and adversely impacted days, underscoring the relationship between mental health and daily functioning [25,26]. Overall, members evidenced significant improvements in reported unhealthy mental health days and adversely impacted days over the month. Furthermore, improvements in reported adversely impacted days were significant for both subclinical members and clinical members, and improvements in reported unhealthy mental health days were significant for clinical members. Our results also found that clinical sessions, but not coaching sessions, predicted changes in reported unhealthy mental health days over the month. Taken together, this study offers preliminary descriptives on a valuable but less commonly used outcome measure, specifically in a traditionally understudied but increasing population of individuals seeking out virtual care. The study further supports how virtual care is a promising strategy to meet the growing demand of mental health services.

Not all individuals seeking out care exceeded industry clinical thresholds. Thus, additional outcome measures, such as the Healthy Days measure, are needed to evaluate the effects of digital mental health care beyond clinically focused measures (eg, PHQ-9 and GAD-7). To our knowledge, we are one of the first to use the Healthy Days measure within this population (ie, individuals seeking out virtual mental health care). Overall, members reported a reduction of 2.71 unhealthy mental health days. Extrapolating from the Humana data [16], this would be equivalent to a decrease of 27.1 hospital admissions per thousand patients and a potential cost savings of US $42.38 per member per month. Thus, virtual mental health care can be seen as a low-intensity approach to achieve better health outcomes at lower cost [12,13].

Our results found a significant association between the reduction in the number of reported unhealthy mental health days and member engagement with clinical sessions, but not with coaching sessions. Coaching, and even more so text-based coaching, differs fundamentally in their objectives and practices compared to clinical care [27,28]. Little is understood regarding the effects of text-based coaching on mental health outcomes. Our findings suggest that the amount of care needed to drive member improvement might vary between text-based coaching and clinical practices [29]. It is possible that additional time/sessions might be needed for coaching goals to be formed, implemented, and subsequently have an impact on behavioral change via a text-based medium [28-30]. Future studies should extend the follow-up window when evaluating coaching sessions and assess alternate trajectories of improvement in mental health (eg, nonlinear).

### Limitations

There are several limitations to consider. One limitation is the potential for bias in our estimates and the increased likelihood that our results may not generalize to all individuals who engage with teletherapy. Furthermore, our cohort design did not have a comparison group or random assignment to the treatment intervention. Thus, our ability to draw causal inferences is limited and improvements in reported unhealthy days could simply be due to a passage of time; however, we were able to demonstrate significant changes in members with both subclinical and clinical symptoms using real-world longitudinal data. Even though data were missing at random and may not bias results, future studies should implement procedures (eg, incentives) to encourage and capture more complete follow-up data. Future studies can also examine obstacles and facilitators for engagement in teletherapy. The study was also limited to available self-reported outcome data, and there was a large amount of attrition in members reporting unhealthy days over time. This could be due to most members not experiencing clinically meaningful baseline symptomatology and potentially quick improvements in functioning. It is also possible that because the survey was administered outside of the Ginger platform (ie, Survey Monkey), the additional step of completing the measure might have been an added time burden. However, this approach allowed us to pilot and demonstrate the real-world attrition rate when using external data collection platforms.

### Conclusions

To our knowledge, this study is one of the first to use the HRQoL measure as a primary outcome in an evaluation of a digital behavioral health platform. Using real-world longitudinal data, our preliminary yet promising results show that short-term engagement with virtual care can be an effective means to improve HRQoL for members with subclinical and clinical symptoms. Virtual care represents a scalable and well-suited approach to meet the growing need for mental health services that has outpaced the in-person availability of clinical mental health professionals. Future studies should examine the long-term impact of text-based coaching and clinical support on HRQoL.

## Abbreviations

CDC: Centers for Disease Control and Prevention
DSM-IV: Diagnostic and Statistical Manual of Mental Disorders (Fourth Edition)
GAD-7: General Anxiety Disorder-7
HRQoL: health-related quality of life
PHQ-9: Patient Health Questionnaire-9
